# Accuracy of a new paired imaging technique for position correction in whole breast radiotherapy

**DOI:** 10.1120/jacmp.v16i1.4796

**Published:** 2015-01-08

**Authors:** Saskia Petillion, Karolien Verhoeven, Caroline Weltens, Frank Van den Heuvel

**Affiliations:** ^1^ Department of Radiation‐Oncology University Hospitals of Leuven KU Leuven Leuven Belgium; ^2^ Gray Institute for Radiation Oncology and Biology University of Oxford Oxford UK

**Keywords:** breast radiotherapy, paired imaging, kV‐MV imaging, kV‐kV imaging

## Abstract

Image‐guided position verification in breast radiotherapy is accurately performed with kilovoltage cone beam CT (kV‐CBCT). The technique is, however, time‐consuming and there is a risk for patient collision. Online position verification performed with orthogonal‐angled mixed modality paired imaging is less time‐consuming at the expense of inferior accuracy compared to kV‐CBCT. We therefore investigated whether a new tangential‐angled single modality paired imaging technique can reduce the residual error (RE) of orthogonal‐angled mixed modality paired imaging. The latter was applied to 20 breast cancer patients. Tangential‐angled single modality paired imaging was investigated in 20 breast and 20 breast cancer patients with locoregional lymph node irradiation. The central lung distance (CLD) residual error and the longitudinal residual error were determined during the first 5 treatment fractions. Off‐line matching of the tangential breast field images, acquired after online position correction, was used. The mean, systematic, and random REs of each patient group were calculated. The systematic REs were checked for significant differences using the F‐test. Tangential‐angled single modality paired imaging significantly reduced the systematic CLD residual error of orthogonal‐angled mixed modality paired imaging for the breast cancer patients, from 2.3 mm to 1.0 mm, and also significantly decreased the systematic longitudinal RE from 2.4 mm to 1.3 mm. PTV margins, which account for the residual error $\left({\text{PTV}}_{\text{RE}}\right)$, were also calculated. The ${\text{PTV}}_{\text{RE}}$ margin needed to account for the RE of orthogonal‐angled mixed modality paired imaging (i.e., 8 mm) was halved by tangential‐angled single modality paired imaging. The differences between the systematic REs of tangential‐angled single modality paired imaging of the breast cancer patients and the breast cancer patients with locoregional lymph node irradiation were not significant, yielding comparable ${\text{PTV}}_{\text{RE}}$ margins. In this study, we showed that tangential‐angled single modality paired imaging is superior to orthogonal‐angled mixed modality paired imaging to correct the position errors in whole breast radiotherapy.

PACS numbers: 87.57N‐, 87.56Da, 87.53Kn

## I. INTRODUCTION

Postoperative whole breast radiotherapy (WBRT) with two wedged, tangential fields is gradually being replaced by more conformal techniques: intensity‐modulated radiotherapy (IMRT), step‐and‐shoot IMRT, simultaneous integrated boost (SIB) radiotherapy or partial breast radiotherapy. Recently, the results of the EORTC trial 22922‐10925^(1)^ have revealed that overall, disease‐free and metastases‐free survival in patients with stage I‐III breast cancer is improved by adding locoregional lymph node (LN) irradiation to WBRT (WBRT‐LN). To maximally spare the organs at risk (OARs) — being the heart and the ipsilateral lung — while maintaining high probability of target coverage, conformal respiratory‐gated^(2)^ techniques are applied.

High position accuracy is vital to prevent the underdosage of the target volumes and the overdosage of the healthy tissues in conformal (respiratory‐gated) WBRT‐LN. Tangential megavoltage (MV) imaging (i.e., MV imaging in the direction of the treatment beam) as applied for 2D position verification in tangential WBRT,^3^, ^4^, ^5^ should not be extrapolated to conformal WBRT‐LN to avoid the geographical miss of the target volume. A technique for accurate 3D position correction is needed. From studies in partial breast radiotherapy^6^, ^7^ and SIB radiotherapy,^(8)^ it is known that 3D position verification is accurately performed with kilovoltage cone‐beam CT (kV‐CBCT).^6^, ^7^, ^8^ However, kV‐CBCT acquisition, reconstruction, and online matching are time‐consuming and there is a risk for patient collision. kV‐CBCT acquisition can be approximated with tomosynthesis^9^, ^10^ or with three electronic portal images,^(11)^ Both techniques are time‐consuming, particularly in respiratory‐gated WBRT(‐LN). One study has reported on the use of almost orthogonal kV localization fields for position verification in SIB radiotherapy.^(12)^ However, the methodology still uses kV‐CBCT to exclude the influence of clip migration,^(13)^ Online position verification with two paired localization fields has been described,^7^, ^12^, ^14^, ^15^ but cannot correct all position errors, yielding residual errors (REs). Fatunase et al,^(7)^ have shown that PTV margins of 9 mm, 7 mm, and 7 mm are needed to account for the vertical, lateral, and longitudinal components of the RE, respectively, in partial breast radiotherapy. Hence, the LN PTV margin prescribed by the EORTC 22922‐10925 trial^(1)^ (i.e., 5 mm) does not compensate for these REs.

The REs of paired imaging‐based position correction in WBRT and WBRT‐LN are, however, unexplored. Visualizing relevant matching anatomy by appropriate localization field orientation and/or beam modality selection should restrict the REs. The purpose of this study was therefore 1) to investigate whether a new tangential‐angled single modality paired imaging technique is superior to our clinically used orthogonal‐angled mixed modality paired imaging technique for position correction in WBRT, and 2) to assess the accuracy of the new technique for position correction in WBRT‐LN.

## II. MATERIALS AND METHODS

### A. Patients and simulation protocol

Sixty patients were included in the study. Forty patients underwent WBRT and 20 patients WBRT‐LN (i.e., WBRT with internal mammary‐medial supraclavicular (IM‐MS) lymph node (LN) irradiation). Simulation of both treatments was identical. The patient was scanned in the treatment position: supine on a breast board, with the arms extended in a support above the head and with an immobilization wedge under the knees. A free‐breathing CT acquisition was performed (Somatom Sensation Open; Siemens Medical Solutions, Erlangen, Germany) and reconstructed with 3 mm slice thickness. To obtain reproducible patient positioning, four points were marked using radiopaque ball bearings prior to CT scanning and tattooed afterwards. Three points were in a transversal plane at half the height of the clinical breast volume: one on the sternum and one on each lateral side of the patient a couple of centimeters above the breast board. The fourth point was marked 15 cm caudal with respect to the sternum point along the sagittal laser line and used for longitudinal patient alignment.

### B. Treatment plans

For each treatment, a single isocentric field setup was used: WBRT with two tangential half‐beams, and WBRT‐LN either with a four‐field oblique parasternal photon technique^(16)^ or a three‐field partly wide tangential technique.^(17)^ The latter techniques share the use of two tangential breast quarter‐beams and a nearly anterior half‐beam for MS LN irradiation (i.e., the MS‐field). Irrespective of the field setup, the PTV margins prescribed in the EORTC 22922‐10925 trial^(1)^ were applied (i.e., 10 mm around the breast and 5 mm around the LNs). The treatments were delivered on a Varian Clinac 2100C/D or a Varian TrueBeam linear accelerator, both equipped with an amorphous silicon electronic portal image detector (EPID), aSi1000, and an On‐Board Imaging system (Varian Medical Systems, Inc., Palo Alto, CA).

### C. Online position verification and correction

The daily patient positioning consisted of the alignment of the tattoos with the treatment room lasers, followed by an isocenter shift. The patient position was verified by online paired matching of two orthogonal localization fields. First, the orthogonal‐angled mixed modality paired imaging technique was investigated on 20 consecutive breast cancer patients. Next, the proposed tangential‐angled single modality paired imaging technique was applied to a second group of 20 consecutive breast cancer patients and to 20 consecutive breast cancer patients with locoregional LN irradiation.

#### C.1 Orthogonal‐angled mixed modality paired imaging (orthogonal kV‐MV imaging)

**Figure 1:**
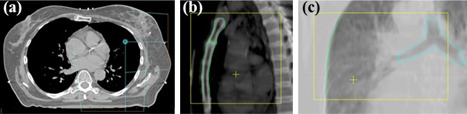
Orthogonal kV‐MV imaging in left‐sided WBRT: setup (a) of the lateral (b) and the posterior (c) orthogonal localization fields.

Orthogonal‐angled mixed modality paired imaging consists of a lateral kV image at the irradiated breast side, and a ventro–dorsal MV image (e.g., a left kV image and a posterior MV image for left‐sided breast cancer patients) (Fig. 1). The longitudinal and the vertical positioning errors were determined on the kV image based on a bony anatomy match. The lateral positioning errors were determined using the projection of the thoracic wall, as well as the carina in the MV image. The carina was not used for longitudinal position verification to avoid the influence of its breathing motion. For brevity, this technique will be referred to as orthogonal kV‐MV imaging.

#### C.2 Tangential‐angled single modality paired imaging (tangential kV‐kV imaging)

**Figure 2:**
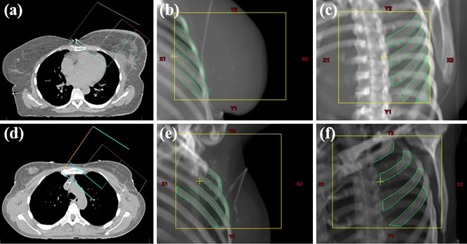
Tangential kV‐kV imaging in left‐sided WBRT (upper row) and in WBRT‐LN (lower row): setup ((a),(d)) of the localization fields in the tangential irradiation direction ((b),(e)) and orthogonal to the irradiation direction ((c),(f)).

Tangential‐angled single modality paired imaging consists of akV image acquired in the direction of one of the tangential fields and an orthogonal kV image. For left‐sided breast cancer patients, the kV image acquisition starts in the direction of the medial field (Fig. 2) and for right‐sided patients in the direction of the lateral tangential field. The ribs close to the target volume were used to align the digitally reconstructed radiographs (DRRs) with the corresponding kV image. First, the tangential field kV image was matched by translating in all necessary directions. Next, the second kV image was matched. Only left–right DRR translations were allowed to prevent matching on a wrong rib. For brevity, this technique will be referred to as tangential kV‐kV imaging.

#### C.3 Correction of positioning errors

For both imaging protocols, online paired matching was performed using a pseudo 3D technique in which a matching template, drawn on the matching anatomy of the DRR, was aligned with the acquired image. After online matching, all translational differences were corrected by applying the appropriate couch shifts prior to the treatment. The rotational error was not measured.

### D. Assessment of the residual error

After position correction, we verified the new patient position by MV imaging of both tangential fields during the first 5 treatment fractions. Moreover, in case of WBRT‐LN, the MS field was kV imaged, taking advantage of the superior visibility of the bony anatomy compared to the MV imaging.

**Figure 3:**
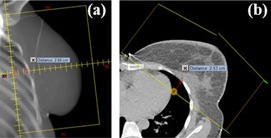
Central lung distance marked on the digitally reconstructed radiograph (a) and on the central axial slice (b).

The difference between the planned treatment field position, shown on the DRR, and the actual treatment field position, shown on the acquired treatment field image, will be denoted as the residual error (RE). The RE was quantified by aligning the matching template, drawn on the bony anatomy of the DDR, with the treatment field. This was done in Off‐line Review (Varian Medical Systems) yielding a residual error vector in the vertical $\left({\text{RE}}_{\text{Vrt}}\right)$, the longitudinal $\left({\text{RE}}_{\text{Lgn}}\right)$, and the lateral $\left({\text{RE}}_{\text{Lat}}\right)$ direction. Rotation of the template was allowed, yielding the rotational error. ${\text{RE}}_{\text{Vrt}}$ and ${\text{RE}}_{\text{Lat}}$ were used to calculate ${\text{RE}}_{\text{CLD}}$ (i.e., the residual error of the central lung distance (CLD)) using the Pythagorean theorem. The CLD is the distance between the deep field edge and the interior chest wall at field central axis^(4)^ (Fig. 3). In case of WBRT‐LN, ${\text{RE}}_{\text{Lat}}$ and ${\text{RE}}_{\text{Lgn}}$ of the MS field were also quantified. ${\text{RE}}_{\text{Vrt}}$ was irrelevant due to the nearly anterior setup of the MS field.

The CLD residual errors and the longitudinal residual errors yielded patient mean residual errors $({\text{RE}}_{\text{CLD},\text{Mean}}$ and ${\text{RE}}_{\text{Lgn},\text{Mean}}$, respectively) and patient random residual errors (1 SD). Next, the population mean residual errors, the systematic residual errors, and the random residual errors were calculated. Each systematic residual error was calculated as the SD of the patient mean residual errors and each random residual error as the root mean square of the patient random residual errors. As the systematic error has much larger impact on the PTV margin than the random error (see Materials & Methods section E), reducing the systematic error is most important. Therefore, we checked the differences between the systematic residual errors of orthogonal kV‐MV and tangential kV‐kV imaging in WBRT and between the residual errors of WBRT and WBRT‐LN after tangential kV‐kV imaging for statistical significance using the F‐test. The differences were considered significant if p≤0.05/2 (with Bonferroni correction for multiple testing). Both tangential fields were analyzed, but the results of only one tangential field per patient group were reported.

### E. Methodology for interstudy comparison

This study reports ${\text{RE}}_{\text{CLD}},\;{\text{RE}}_{\text{Lgn}}$, and ${\text{RE}}_{\text{Rot}}$. In contrast, previous studies^6^, ^7^ have reported ${\text{RE}}_{\text{Vrt}},\;{\text{RE}}_{\text{Lgn}}$, and ${\text{RE}}_{\text{Lat}}$. Due to the different approach, straightforward interstudy comparison is only possible for ${\text{RE}}_{\text{Lgn}}$. Comparison of ${\text{RE}}_{\text{CLD}}$ with ${\text{RE}}_{\text{Vrt}}$ and ${\text{RE}}_{\text{Lat}}$ is possible by calculation of the PTV margin needed to account for the residual error (${\text{PTV}}_{\text{RE}}$) using a margin recipe of van Herk et al.:.^(18)^ ${\text{PTV}}_{\text{RE}}=2.5\Sigma_{\text{RE}}+0.7\sigma_{\text{RE}}$, with $\Sigma_{\text{RE}}$ the systematic and $\sigma_{\text{RE}}$ the random residual error. The formula assumes perfect 3D dose conformation, which is not the case in WBRT and WBRT‐LN using opposed tangential beams, as in this study. The parameters of 2D dose conformation are slightly smaller than those of 3D dose conformation,^(18)^ However, the ${\text{PTV}}_{\text{RE}}$ should be used as a lower limit for the ${\text{PTV}}_{\text{RE}}$ margin as it does not take into account the rotational errors.

## III. RESULTS

### A. Comparison of the residual errors of orthogonal kV‐MV and tangential kV‐kV imaging in WBRT

**Figure 4:**
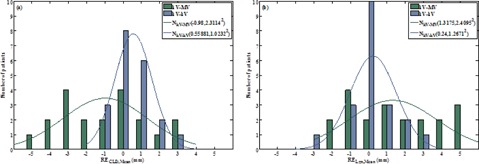
Distribution of the patient mean CLD residual error (${\text{RE}}_{\text{CLD},\text{Mean}}$ (a)) and the patient mean longitudinal residual error (${\text{RE}}_{\text{Lgn},\text{Mean}}$ (b)) after orthogonal kV‐MV or tangential kV‐kV imaging in WBRT. $N(\mu ,\Sigma^{2})=\text{fitted Gaussian curve with mean}\;(\mu )\;\text{and SD}\;(\Sigma )$.

Figure 4 shows the distribution of the patient mean CLD and longitudinal residual errors of orthogonal kV‐MV and tangential kV‐kV imaging. In both, the smallest 95% confidence interval was found for tangential kV‐kV imaging (Table 1). Orthogonal kV‐MV imaging was stopped after 5 fractions if the patient's mean residual error was unacceptable (i.e., ≥4 mm). Tangential kV‐kV imaging could be continued for all patients.

From Table 1, we note that tangential kV‐kV imaging significantly $\left(p=8.10^{-4}\right)$ decreased the systematic CLD residual error compared to orthogonal kV‐MV imaging. Likewise, a comparable, statistically significant (p=0.007), decrease was found in the systematic longitudinal residual error component. A ${\text{PTV}}_{\text{RE},\text{CLD}}$ and ${\text{PTV}}_{\text{RE},\text{Lgn}}$ of 7 mm and 8 mm, respectively, accounted for the residual error of orthogonal kV‐MV imaging which were both reduced to 4 mm with tangential kV‐kV imaging.

As the rotational errors were not corrected, both protocols had comparable rotational errors (Table 2).

**Table 1:** 95% confidence interval (CI), mean $\left(\mu_{\text{RE}}\right)$, systematic $\left(\Sigma_{\text{RE}}\right)$, and random $\left(\sigma_{\text{RE}}\right)$ residual errors (REs) of orthogonal kV‐MV imaging in WBRT, tangential kV‐kV imaging in WBRT, and tangential kV‐kV imaging in WBRT‐LN. The differences between the systematic residual errors of orthogonal kV‐MV and tangential kV‐kV imaging in WBRT were statistical significant (F‐test, $p\leq 0.05/2^{a}$). The differences between the systematic residual errors of tangential kV‐kV imaging of both breast cancer treatments were not statistically significant (F‐test, p>0.05/2). ${\text{PTV}}_{\text{RE}}$ is the PTV margin which accounts for the residual errors

	*WBRT* (N=20) *kV‐MV*		*WBRT* (N=20) *kV‐kV*		*WBRT‐LN* (N=20) *kV‐kV*	
	${\text{RE}}_{\text{CLD}}$	${\text{RE}}_{\text{Lgn}}$	${\text{RE}}_{\text{CLD}}$	${\text{RE}}_{\text{Lgn}}$	${\text{RE}}_{\text{CLD}}$	${\text{RE}}_{\text{Lgn}}$
95% CI (mm)	[−2.1,0.1]	[0.2,2.4]	[0.08,1.0]	[−0.4,0.8]	$\left[-4.10^{-3},0.7\right]$	[0.4,1.2]
$\mu_{\text{RE}}\;(\text{mm})$	−0.9	1.3	0.6	0.2	0.4	0.8
$\Sigma_{\text{RE}}\;(\text{mm})$	2.3	2.4	1.0^a^	1.3^a^	0.8	0.9
$\sigma_{\text{RE}}\;(\text{mm})$	2.1	3.1	1.9	1.7	1.2	1.2
${\text{PTV}}_{\text{RE}}\;(\text{mm})$	7	8	4	4	3	3

**Table 2:** 95% confidence interval (CI), mean (µ), systematic (Σ), and random (σ) rotational errors in WBRT and in WBRT‐LN

	*WBRT* (N=20) *kV‐MV*	*WBRT* (N=20) *kV‐kV*	*WBRT‐LN* (N=20) *kV‐kV*
95% CI (mm)	[−0.5,0.7]	[−0.4,0.5]	$\left[2.10^{-3},0.7\right]$
μ (mm)	0.09	0.03	0.3
Σ (mm)	1.2	0.9	0.7
σ (mm)	0.8	0.6	0.6

### B. Assessment of the residual errors of tangential kV‐kV imaging in WBRT‐LN

**Figure 5:**
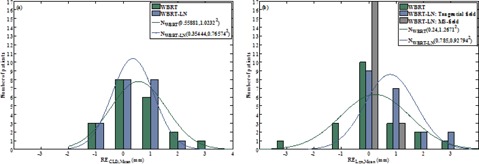
Distribution of the patient mean CLD residual error (${\text{RE}}_{\text{CLD},\text{Mean}}$ (a)) and the patient mean longitudinal residual error (${\text{RE}}_{\text{Lgn},\text{Mean}}$ (b)) after tangential kV‐kV imaging in WBRT and WBRT‐LN. $N(\mu ,\Sigma^{2})=\text{fitted Gaussian curve with mean}\;(\mu (\;\text{and SD}\;)\Sigma )$.

Due to the superior results of tangential kV‐kV imaging in WBRT, its residual errors in WBRT‐LN were also determined. Figure 5 shows the distributions of the patient mean CLD and longitudinal residual errors of tangential kV‐kV imaging in WBRT‐LN and WBRT. In case of WBRT‐LN, the longitudinal residual error was measured both in the tangential field and in the MS field. The residual error of the latter confirmed the residual error of the tangential field.

The systematic CLD, as well as the systematic longitudinal residual errors of tangential kV‐kV imaging in WBRT‐LN and in WBRT, were not significantly different (p=0.2) (Table 1). The 3D PTV margin needed to compensate for the residual errors in WBRT‐LN amounted 3 mm, which compared well to the 3D ${\text{PTV}}_{\text{RE}}$ margin of WBRT (Table 1).

As expected, due to uncorrected rotational errors, both breast treatments had comparable rotational errors (Table 2).

## IV. DISCUSSION

In this study we were able to show that the residual errors of the new tangential‐angled single modality (kV‐kV)‐based position correction protocol are significantly smaller than those of the previously used orthogonal‐angled mixed modality (kV‐MV)‐based position correction protocol. Moreover, we found that the residual errors of the former show no significant difference between WBRT and WBRT with locoregional lymph node irradiation.

We found that PTV margins of 7 mm and 8 mm accommodate for the CLD and longitudinal residual errors, respectively, of orthogonal kV‐MV imaging. A study by Fatunase et al.^(7)^ confirms our PTV margins. Using their reported results, we could estimate the patient mean vertical, longitudinal, and lateral residual errors and calculate the corresponding systematic and random residual errors. Their systematic and random longitudinal residual errors, 2.0 mm and 3.1 mm respectively, yielded a ${\text{PTV}}_{\text{RE},\text{Lgn}}$ of 7 mm. Their systematic and random vertical residual errors (2.5 mm and 3.6 mm, respectively) and systematic and random lateral residual errors (1.9 mm and 3.2 mm, respectively) yielded a ${\text{PTV}}_{\text{RE},\text{Vrt}}$ of 9 mm and a ${\text{PTV}}_{\text{RE},\text{Lat}}$ of 7 mm, well corresponding to our ${\text{PTV}}_{\text{RE},\text{CLD}}$ value of 7 mm. The large residual errors of orthogonal kV‐MV imaging are due to the thoracic wall and the carina being a bad surrogate for the breast. The distance between the matching anatomy and the isocenter, in combination with the divergence of the ionizing beams, causes an under‐ or overestimation of the real position error.^(19)^

These results confirmed that orthogonal kV‐MV imaging had no future for position verification in breast radiotherapy. Contralateral breast irradiation to visualize the carina shadow in the MV image with low resulting position accuracy is ethically unacceptable. A more relevant surrogate for the target volume needed to be visualized in the localization fields (e.g., the ribs). Changing the beam orientation to perform tangential kV‐MV imaging complicated and prolonged online matching due to the inferior visibility of the ribs in the tangential MV image. This pilot study was therefore stopped without quantifying the residual errors. Tangential kV‐MV imaging was replaced by tangential kV‐kV imaging for further investigation. The systematic and random longitudinal residual errors of the new tangential‐angled kV‐kV‐based correction protocol (1.3 mm and 1.7 mm, respectively) compare well to the longitudinal residual errors of 3D kV‐CBCT–guided patient position correction. White et al.^(6)^ found a systematic and random longitudinal residual error of 0.7 mm and 1.4 mm, respectively. Moreover, the ${\text{PTV}}_{\text{RE},\text{CLD}}$ we calculated for tangential kV‐kV imaging in WBRT, 4 mm, is comparable to the ${\text{PTV}}_{\text{RE},\text{Vrt}}$ and ${\text{PTV}}_{\text{RE},\text{Lat}}$ found by White et al.^(6)^ — 4 mm and 3 mm, respectively. The 3D similarity between tangential kV‐kV and kV‐CBCT imaging was expected as the matching structures (i.e., the ribs) were selected in analogy with other kV‐CBCT studies in WBRT.^8^, ^20^

The appropriateness of the ribs as matching structures for online position verification in WBRT has previously been shown. Several studies^5^, ^21^, ^22^ concluded that the interfraction CLD variation (due to patient setup error) is more significant than the intrafraction CLD variation (due to patient and respiratory motion). As an example, Fein et al.^(21)^ reported an intrafraction CLD variation (1 SD) of 1.6 mm compared to an interfraction CLD variation (1 SD) of 4.4 mm. Due to the randomness of the breathing motion, its influence is incorporated in our random residual errors. After tangential kV‐kV imaging, all random residual errors were ≤1.9 mm. We also analyzed the portal images of the second tangential field (results not shown). The longest time interval between the acquisitions of both tangential images was recorded in WBRT‐LN, making the technique more sensitive to the patient and respiratory motion. However, small mean registration differences between both tangential images were found — 0.6 mm for the CLD and 0.1 mm in longitudinal direction. Topolnjak et al.^(8)^ have reported comparable mean registration differences (≤0.6 mm).

An online position correction protocol with a minimal residual error is important in order to limit the PTV margin, in particular in WBRT‐LN. The applied 10 mm breast PTV margin accounts for the maximum ${\text{PTV}}_{\text{RE}}$ of orthogonal kV‐MV imaging (8 mm). Hence, as reported by Fatunase et al.,^(7)^ the residual error has only modest clinical relevance in WBRT. In contrast, an IM‐MS PTV margin of 5 mm, prescribed by the EORTC trial 22922‐10925,^(1)^ is insufficient to account for the residual errors of orthogonal kV‐MV imaging. Doubling the IM‐MS PTV margin is prevented by the OAR dose. For example, Erven et al.^(23)^ have shown that, in case of left‐sided WBRT‐LN with 10 mm breast and 5 mm IMMS PTV margins, heart volumes >10% receive doses ≥30 Gy. This violates the heart constraint proposed by Marks et al.^(24)^ of $V_{30\text{Gy}}\leq 10\%$. Erven and colleagues propose deep inspiration breath‐hold radiation therapy as the preferred technique, to significantly reduce the heart volumes receiving ≥30 Gy. However, doubling the IM‐MS PTV margin will challenge deep inspiration breath‐hold radiotherapy to meet the heart constraint for all patients. The PTV margins prescribed in the EORTC trial 22922‐10925 can be retained by applying tangential kV‐kV imaging. Daily tangential kV‐kV imaging even reduces the EORTC PTV margins to an isotropic 3 mm PTV margin for both target volumes. Daily application of the tangential kV‐kV technique is also preferred for young breast cancer patients as, during image acquisition, mainly the target volumes are irradiated and the healthy tissue is maximally spared. Moreover, the kV‐kV imaging dose is lower than the kV‐MV imaging dose.

Except for its accuracy and its appropriateness in WBRT and WBRT‐LN, tangential kV‐kV imaging has additional important properties. 1) The procedure is as time efficient as orthogonal kV‐MV imaging. The sequence of the kV image acquisition can be chosen such that, after position correction, no gantry rotation is needed before the irradiation can be started (Fig. 2). It is less time‐consuming then kV‐CBCT position verification which requires a gantry rotation of at least 180° during image acquisition, image reconstruction, loading of the planning CT, and gantry rotation to the first irradiation angle. 2) Tangential kV‐kV imaging is easily extendable to respiratory‐gated breast radiotherapy. A disadvantage of tangential kV‐kV imaging is that it cannot correct rotational errors. We found small systematic rotational errors $\left(\leq 1.2^{\circ }\right)$ which are comparable to previously reported systematic rotational errors of 2°.^(25)^ Thanks to their smallness, the rotational errors do not influence the accuracy of kV‐kV imaging.

A limitation of the study is the semi‐3D residual error determination by the tangential MV imaging: (${\text{RE}}_{\text{CLD}}$ and ${\text{RE}}_{\text{Lgn}}$) instead of (${\text{RE}}_{\text{Vrt}}$, ${\text{RE}}_{\text{Lgn}}$, and ${\text{RE}}_{\text{Lat}}$). Although tangential MV imaging is insufficient for accurate 3D position verification, it is still useful for our comparative study on the residual errors of different online position correction protocols. It focuses on the residual error components with most clinical impact in tangential beam breast radiotherapy (${\text{RE}}_{\text{CLD}}$ and ${\text{RE}}_{\text{Lgn}}$). The residual error in the direction of the tangential beam can be neglected as it has small dosimetric impact due to the opposing field setup used in WBRT and WBRT‐LN. This semi‐3D residual error determination was preferred to kV‐CBCT acquisition: 1) to minimize the imaging dose (treatment field images are acquired in clinical routine for field edge and leaf position verification), and 2) to determine the accuracy of the field alignment after pseudo 3D online position correction in WBRT.

Online tangential kV‐kV imaging is clinically used in our department for position correction in WBRT and WBRT‐LN. MV imaging of the treatment fields is restricted to the first treatment fraction for field edge verification. In the future, tangential‐angled kV‐kV–based position correction will be extended to respiratory‐gated WBRT and respiratory‐gated WBRT‐LN. Tangential kV‐kV imaging will also be used to investigate the radiobiological impact of systematic patient position errors and their correction in WBRT‐LN. The results will be discussed in an upcoming study.

## V. CONCLUSIONS

The new tangential‐angled single modality (kV‐kV) paired imaging technique is superior to the orthogonal‐angled mixed modality (kV‐MV) paired imaging technique for position correction in WBRT. Moreover, comparable position correction accuracy is obtained in WBRT‐LN.

## ACKNOWLEDGMENTS

The authors would like to express their appreciation to Prof. Dr. Peeters, Prof. Dr. Janssen, and Prof. Dr. Van Limbergen for their comments and suggestions. We would also like to thank the Myny‐Vanderpoorten Foundation for fnancial support.
